# In-Vivo Gene Signatures of *Mycobacterium tuberculosis* in C3HeB/FeJ Mice

**DOI:** 10.1371/journal.pone.0135208

**Published:** 2015-08-13

**Authors:** Uma Shankar Gautam, Smriti Mehra, Deepak Kaushal

**Affiliations:** 1 Tulane National Primate Research Center, Covington, Louisiana, United States of America; 2 Louisiana State University School of Veterinary Medicine Department of Pathobiological Sciences, Baton Rouge, Louisiana, United States of America; 3 Microbiology and Immunology, Tulane University School of Medicine, New Orleans, Louisiana, United States of America; Public Health Research Institute at RBHS, UNITED STATES

## Abstract

Despite considerable progress in understanding the pathogenesis of *Mycobacterium tuberculosis* (*Mtb*), development of new therapeutics and vaccines against it has proven difficult. This is at least in part due to the use of less than optimal models of *in-vivo Mtb* infection, which has precluded a study of the physiology of the pathogen in niches where it actually persists. C3HeB/FeJ (Kramnik) mice develop human-like lesions when experimentally infected with *Mtb* and thus make available, a faithful and highly tractable system to study the physiology of the pathogen *in-vivo*. We compared the transcriptomics of *Mtb* and various mutants in the DosR (DevR) regulon derived from Kramnik mouse granulomas to those cultured *in-vitro*. We recently showed that mutant Δ*dosS* is attenuated in C3HeB/FeJ mice. Aerosol exposure of mice with the mutant mycobacteria resulted in a substantially different and a relatively weaker transcriptional response (< = 20 genes were induced) for the functional category ‘Information Pathways’ in *Mtb*:Δ*dosR*; ‘Lipid Metabolism’ in *Mtb*:Δ*dosT*; ‘Virulence, Detoxification, Adaptation’ in both *Mtb*:Δ*dosR* and *Mtb*:Δ*dosT*; and ‘PE/PPE’ family in all mutant strains compare to wild-type *Mtb* H37Rv, suggesting that the inability to induce DosR functions to different levels can modulate the interaction of the pathogen with the host. The *Mtb* genes expressed during growth in C3HeB/FeJ mice appear to reflect adaptation to differential nutrient utilization for survival in mouse lungs. The genes such as *glnB*, Rv0744c, Rv3281, *sdhD/B*, *mce4A*, *dctA* etc. downregulated in mutant Δ*dosS* indicate their requirement for bacterial growth and flow of carbon/energy source from host cells. We conclude that genes expressed in *Mtb* during *in-vivo* chronic phase of infection in Kramnik mice mainly contribute to growth, cell wall processes, lipid metabolism, and virulence.

## Introduction

Delineating mycobacterial gene expression *in-vivo* is central to the understanding how bacilli invade and interact with or disrupt host cell functions, to facilitate their adaptation to different microenvironments [1–7]. A clear understanding of the molecular events responsible for establishing and maintaining *Mtb* infection is thus essential to develop approaches to contain the disease. However, this requires the use of faithful models of human *Mtb* infection. The traditional mouse model does not result in the formation of human-like granulomas upon experimental infection [8]. For example, C57Bl/6 mice do not faithfully reproduce certain aspects of human TB. In contrast, C3HeB/FeJ mice display lesions with prominent necrotic degeneration, thus more closely resembling human granulomas [9–11]. It has been previously demonstrated that tubercle lesions in C3HeB/FeJ mice develop hypoxia [10–12]. This results in the induction of the DosR regulon that likely enables *Mtb* to persist in hypoxic conditions [13, 14] and within human-like lesions present in the lungs of *Mtb* infected non-human primates [15]. Induction of DosR and the resulting downstream transcriptional changes then likely cause significant perturbation in the metabolic profile of the pathogen. It is postulated that this not only assists the survival of *Mtb* in the changed milieu, allowing it to conserve energy while remaining viable in an anorexic environment, but likely also results in altered antigen presentation [16, 17] and thus adaptive responses [15]. Such changes likely impact significantly, the ability of the antigen-specific responses to control *Mtb* replication and might facilitate the persistence of the pathogen over the long-haul. Therefore, we tested the regulation of *Mtb* genes by comparing the transcription profile that investigated the effects of TB infection in C3HeB/FeJ mice.

Here we report expression profiles of mycobacterial genes upon infection of C3HeB/FeJ mice with *Mtb* H37Rv wild-type (WT) (henceforth referred to as *Mtb*) relative to the mutants defective in response regulator DosR (*Mtb*:Δ*dosR*) or sensor kinases DosS (*Mtb*:Δ*dosS*) or DosT (*Mtb*:Δ*dosT*) during chronic phase of infection in C3HeB/FeJ mice by using DNA microarrays.

## Materials and Methods

### Bacterial Strains and Animals

We used frozen lung samples of C3HeB/FeJ mice from a previous study [11]. The *Mtb* and Dos mutants were revived from frozen stocks and cultured as described [18].

### In-Situ RNA Hybridization

*In-situ* RNA-RNA hybridizations designed to detect mycobacteria specific transcripts on 5 μm section of paraffin-embedded lung tissue (RNase-free) with appropriate controls were performed essentially as described earlier [19].

### Preparation of RNA samples

RNA was isolated from frozen lung samples from our previous study [11]. Frozen tissue (15–20 mg lung lobe of mice) samples were placed in a sterile plastic tissue sample bag, crushed mechanically, transferred to screw caped tube containing 700 μl Qiazol (Qiazen, Germany), followed by mixing the contents and incubated at room temp for 10 minutes. The samples were lysed by bead beating in Lysing Matrix B tubes (MP Biomedicals, USA), added with 140 μl chloroform and mixed by inverting the tubes several time followed by incubation for 5 min at room temp and centrifugation at 13,000 rpm for 15 min at 4°C. RNA was purified with RNA purification kit (Qiazen, Germany) and used in microarrays as described [18]. RNA was isolated from *in-vitro* grown *Mtb* cultures as described [20].

### RNA Quantification and Real Time PCR

The amount of total RNA from *in-vitro* and *in-vivo* samples used for each hybridization was quantified first by RT-PCR as previously described [21]. Toward this, total RNA isolated from lung tissue was subjected to microbe enrichment kit for removal of host RNA followed by microbe express and bacterial RNA amplification, strictly as per manufacturer’s instructions (Life Technologies, USA). RNA isolated from *in-vitro* grown cultures was also treated with microbe express and amplified in parallel. RT-PCR was carried out with cDNA that was reverse transcribed from 1000 ng DNA-free RNA as described [21]. For quantification, a series of genomic DNA with 10-fold dilution was used in RT-PCR as described [21]. Constitutive *sigA* mRNA was quantified in all samples and used as invariable housekeeping control in RT-PCR. The data was normalized to *sigA*. We also tested the levels of *dnaB* in *Mtb* and Dos mutants grown *in-vitro* or those isolated from mouse lung samples.

### DNA Microarrays and Sample hybridization

*Mtb* specific DNA microarrays (MYcroarrays, Biodiscovery Llc, USA) were used to compare transcriptome-wide responses in WT *Mtb* and the Dos mutant strains isolated from mouse lung samples from our previous study [11]. Detailed protocols for array procedures have been described earlier [22]. Differences in the magnitude of gene expression relative to cultures grown till log phase were subjected to statistical analysis using corrected ANOVA (P<0.05) in 2 biological replicate arrays and in every technical replicate spot on each array. Real-time (RT) PCR was performed as previously described [20]. The gene expression levels were normalized to *sig*A. The microarray data has been assigned a GEO (Gene Expression Omnibus) and is publicly available using the accession number GSE70765.

### Comparative transcriptomics

We compared our current data on *Mtb* gene-expression using DNA microarray technology, to that obtained previously from BALB/c mice [7]), macrophages [23]) and *in-vitro* (NRP) conditions [24]). First, we collected all the genes (1.5-fold with increased or decreased expression in datasets obtained from Kramnik mice in the current study and compared these to the datasets (gene expression values in fold-change) obtained during growth in BALB/c mice, macrophages and *in-vitro* (NRP) conditions. The fold-change in gene expression was plotted against mycobacterial genes obtained from various datasets.

## Results

### Detection of RNA in the infected lungs of C3HeB/FeJ mice

**Fig 1:**
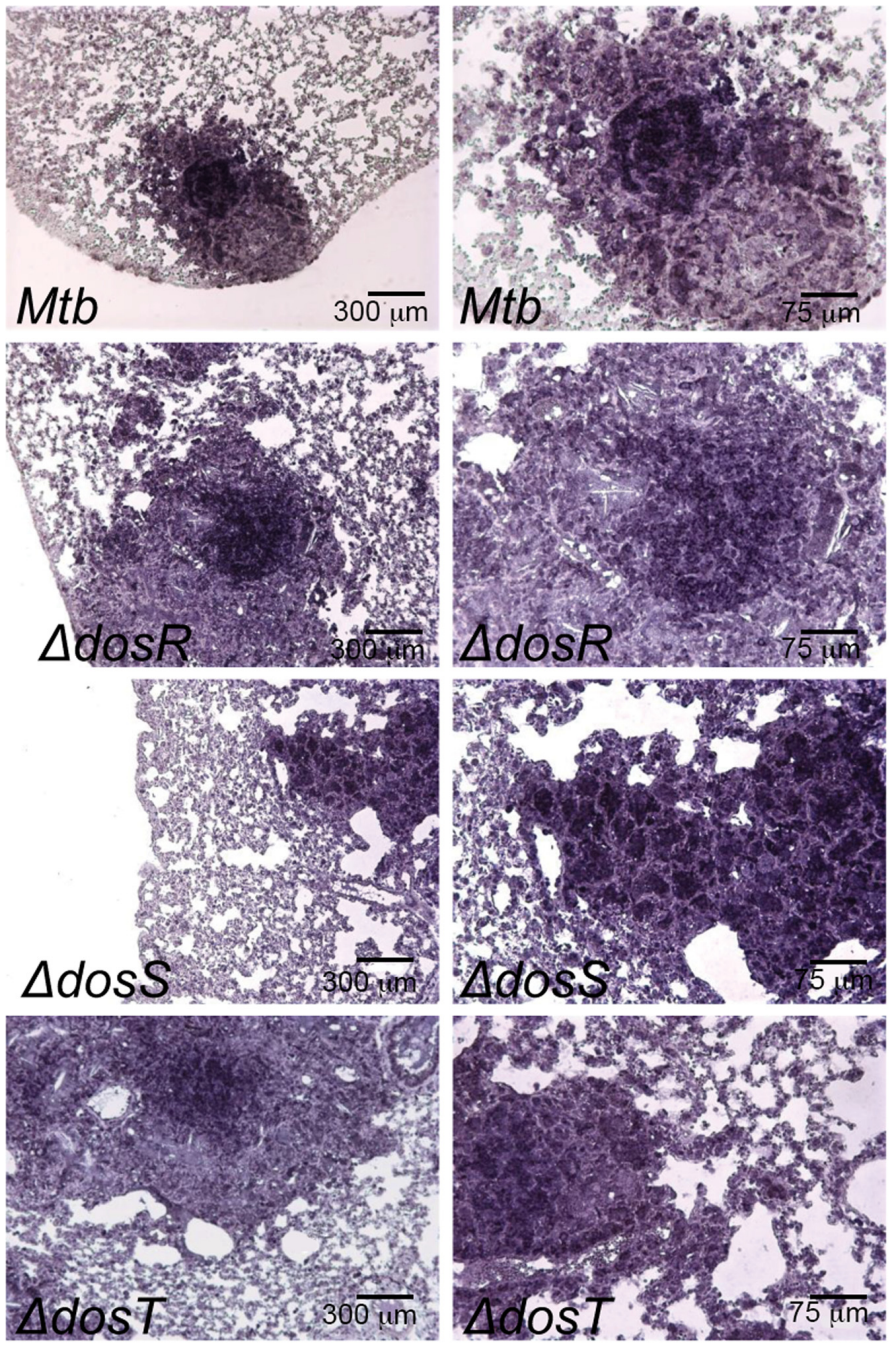
In-Situ hybridization. *In-Situ* hybridization detected the presence of *Mtb* specific *sigA* transcripts in mice lung samples (derived at chronic phase of infection) infected with *Mtb*, *Mtb*:Δ*dosR*, *Mtb*:Δ*dosS* and *Mtb*:Δ*dosT* strains. Representative images with low (left) and high (right) magnification for each *Mtb* strain is shown.

Quantitative RT-PCR on amplified RNA samples derived from *Mtb*-, *Mtb*:Δ*dosR*-, *Mtb*:Δ*dosS-* and *Mtb*:Δ*dosT*-infected mouse lungs at the chronic stage of infection (frozen lung samples were derived from a prior *in-vivo* study [11]) exhibited the presence of transcripts of gene *sigA* (not shown). *sigA* transcripts were also detected by *In-Situ* hybridization using a specific probe generated using the DIG-RNA labeling kit (Roche). Lung granulomas from Kramnik mice infected with *Mtb* and Dos mutants contained abundant *sigA* transcripts as visualized by brown signal (Fig 1), although not all cells stained positive. On the contrary, the expression levels of *dnaB* did not exhibit induced expression, when assayed by RT-PCR (not shown). Together, these two results strongly suggest that *Mtb sigA* mRNA is expressed at high levels in infected lung lesions [25].

### Functional analysis of *Mtb* genes expressed in C3HeB/FeJ mouse lungs

We compared the changes in bacterial gene expression levels by microarray in WT *Mtb* and Dos mutant strains during chronic phase of *in-vivo* growth in C3HeB/FeJ mice. Total RNA was isolated and purified from mouse lung samples as well as from *Mtb* and Dos mutant strains cultured *in-vitro*. Prior to microarray analyses, purified RNA samples were enriched for bacterial mRNA, subjected to amplification and then normalized on the basis of the invariant gene-expression exhibited by *sigA* in both lung and *in-vitro* grown cultures by RT-PCR (not shown). For microarray experiments, RNA was isolated from lung, and profiled relative to the RNA isolated from control samples (*in-vitro* grown *Mtb* and Dos mutant cultures).

**Fig 2:**
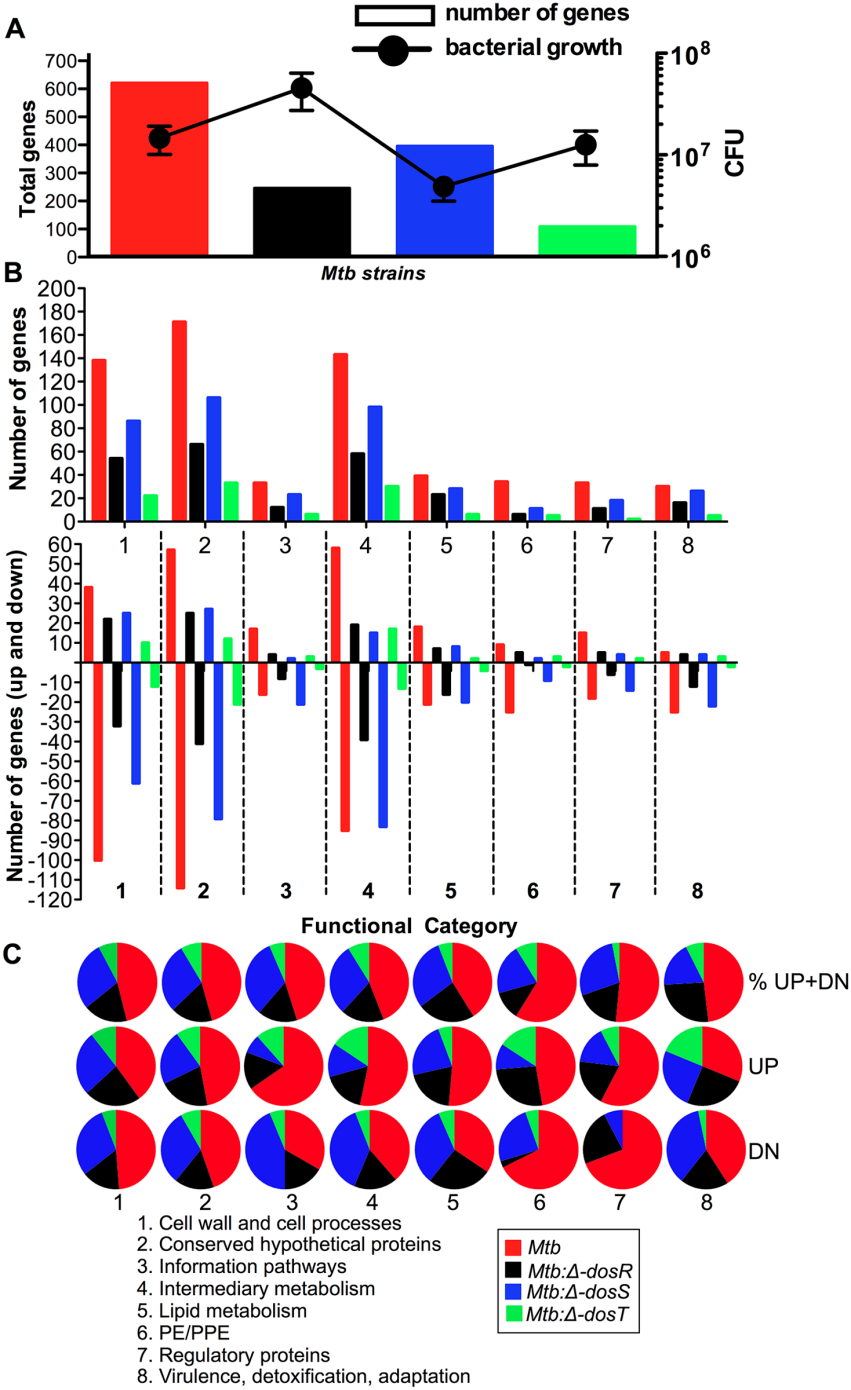
Functional categories with significant changes in gene expression in DNA microarray and *Mtb* growth. A. The graph shows the total number of genes (left) changed in DNA microarray and mycobacterial colony-forming units (CFU) in mouse lungs during *Mtb*, *Mtb*:Δ*dosR*, *Mtb*:Δ*dosS* and *Mtb*:Δ*dosT* infection. B. Functional categories with significant changes in gene expression and number of genes either up or down (cut off 1.5 fold, P<0.05) are shown in each data set. **C**. Percentage of genes (obtained from panels A and B) is shown for each functional category.

**Fig 3:**
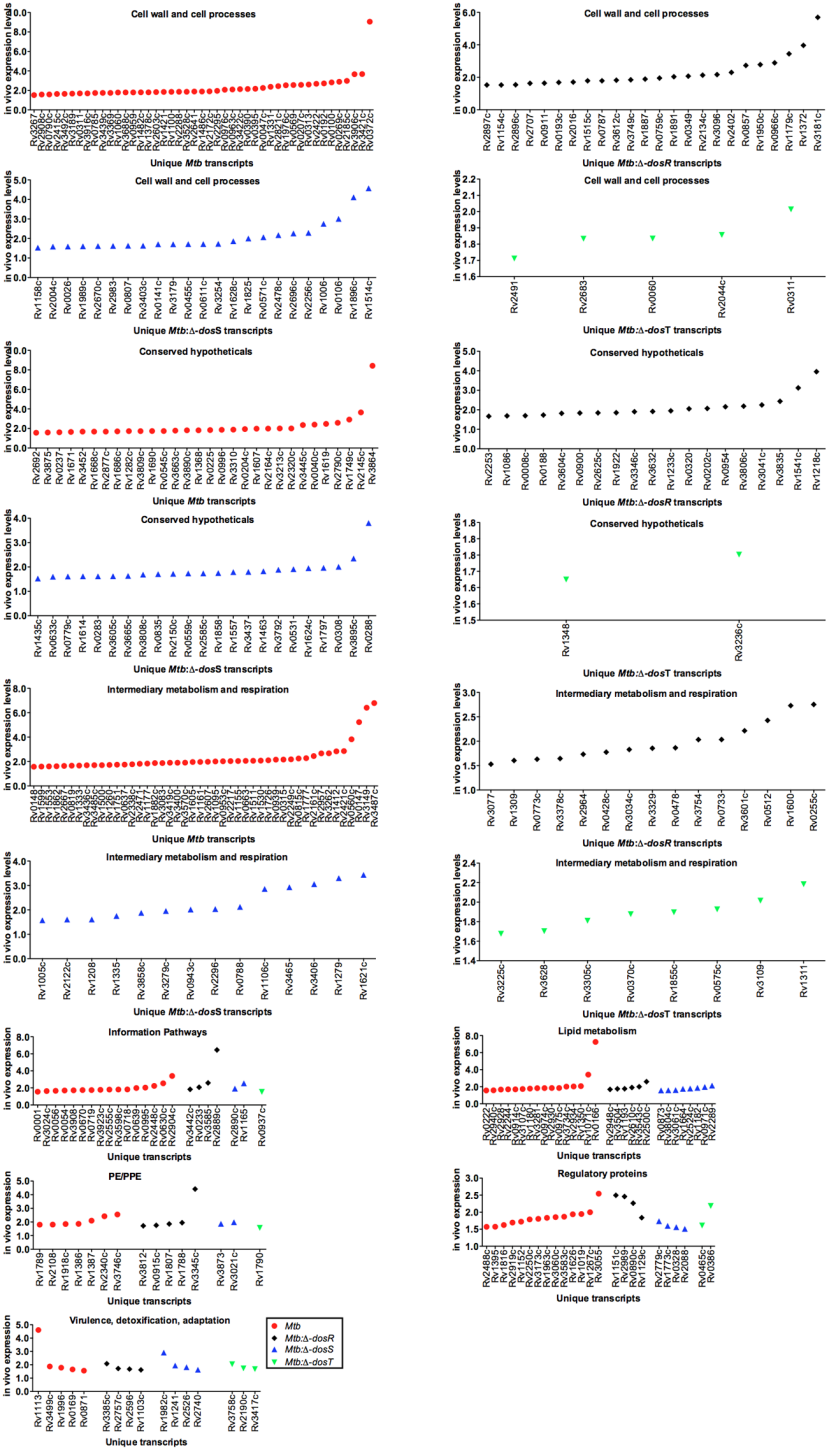
Gene expression in C3HeB/FeJ mouse lungs. The unique genes (on X-axis) expressed (gene expression obtained in microarray using *sigA* normalized RNA of *Mtb* and Dos mutants derived from mouse lungs compared to *in-vitro* grown culture are shown on Y-axis) in C3HeB/FeJ mouse lungs infected with *Mtb* (red circle) or *Mtb*:Δ*dosR* (black diamond) or *Mtb*:Δ*dosS* (blue, upright triangle) or *Mtb*:Δ*dosT* (green, inverted triangle) are shown. Various functional categories are indicated according to the information available in the ‘Tuberculist’ database.

Statistical analyses revealed that a group of 650, 255, 406 and 114 mycobacterial genes whose expression state changed (up-/down-regulated) respectively for each *Mtb*, *Mtb*:Δ*dosR*, *Mtb*:Δ*dosS* and *Mtb*:Δ*dosT* (Fig 2A). We identified several differentially expressed genes between *Mtb* and Dos mutants in mouse lungs, which delineate various functional categories as defined in the “Tuberculist” database (http://tuberculist.epfl.ch) (Fig 2B–2C). The results in Fig 2B–2C were calculated for a given data set, based on the total number of genes assigned to each category in the genome and then compared to the actual number of genes in a functional category induced or repressed for a *Mtb* strain. A group of genes involved in functional category ‘lipid metabolism’, ‘cell wall biosynthesis’ as well as those encoding various ‘regulatory’ and ‘virulence, detoxification, adaptation’ proteins etc. demonstrated statistically significant (P<0.05) differences in gene expression (Fig 3 and S1 Table). Various functional categories have been discussed below.

### Information pathways

DNA recombination and repair result in an increased mutation frequency and better adaptability of the bacterium to stressful conditions inside the host. Thus, genes involved in DNA damage, repair and recombination e.g. Rv3585, Rv0630c etc. changed in all datasets may indicate modification of mycobacterial genes in hostile environment. In addition, a set of genes required for DNA replication, transcription and translation processes were also upregulated; Rv0001, Rv0056, Rv0718, Rv0719, Rv2904 etc. in *Mtb-*; Rv3442c, Rv0233, Rv3585, Rv2889c etc. in *Mtb*:Δ*dosR*-; Rv1165, Rv2890c in *Mtb*:Δ*dosS*-; Rv0937c, Rv3585, Rv0630c in *Mtb*:Δ*dosT*-infected lung samples. The genes *pks6* encoding a polyketide synthase and *tgs2* (Rv0045c) encoding a putative triacylglycerol synthase (diacylglycerol acyltransferase) were also changed in *Mtb* WT, *Mtb*:Δ*dosR* and *Mtb*:Δ*dosT* datasets.

It is known that *Mtb* gene products which are involved in the import of host-derived fatty acids and synthesis of tri-acyl glycerol (TAG) might play critical roles in the energy metabolism during dormant stage [26]. Interestingly, among iVEGI (*In-vivo* Expressed Genomic Island) signature genes as described previously [7] Rv0974c (1.8 fold up), Rv0976c (2.0 fold up) involved in lipid metabolism and Rv0996 (1.83 fold) encoding a transmembrane protein involved in cell wall and cell processes also expressed in mice infected with *Mtb*. On the other hand *fadE12* (Rv0972c, 1.62-fold upregulation) and Rv0992 (2.6-fold upregulation) involved in lipid metabolism and conserved hypothetical protein (unknown function) respectively were also changed in *Mtb* WT-infected lung samples only. In summary, the expression of different genes but those involved in lipid metabolism in all datasets indicate degradation of host-cell lipids is vital in the intracellular life of bacilli and host cells may provide potential precursors for various mycobacterial metabolic processes and cell wall constituents required during growth in C3HeB/FeJ mice. Genes involved in lipid metabolism were examined next.

### Lipid metabolism

In *Mtb*, the expression of *fadD26* (Rv2930), involved in phthioceroldimycocerosate (PDIM) biosynthesis; *fadE13* (Rv0975c), probable acyl-CoA dehydrogenase; *ppsD* (Rv2934) involved PDIM biosynthesis; *fabG2* (Rv1350), *echA9* (Rv071c) involved in the fatty acid biosynthesis and *fadD5* (Rv0166) in lipid degradation was noted. In *Mtb*:Δ*dosR*, *fadD22* (Rv2948c, 1.7 fold up) involved in biosynthesis of phenolic glycolipids (PGLs) and PDIM biosynthesis was upregulated. Similarly following genes involved in lipid metabolism (but different from WT *Mtb* dataset) were specifically demonstrated high expression in *Mtb*:Δ*dosR* infected samples; *fadE26* (Rv3504), *fadD36* (Rv1193) and *fadE29* (Rv3543c) involved in lipid degradation; *fadE19* (Rv2005c), in fatty acids metabolism. In *Mtb*:Δ*dosS*-infected samples following genes of lipid metabolism pathway were expressed; *fadE10* (Rv0873) encoding an acyl-CoA dehydrogenase involved in lipid degradation; *fbpA* (Rv3804c) necessary for cell wall mycolation and biogenesis of trehalose dimycolate (cord factor) and to maintain cell wall integrity; *fadE22* (Rv3061c) probable acyl-CoA dehydrogenase; *pks9* (Rv1664) probable polyketide synthase; *fas* (Rv2524c) probable fatty acid synthase (fatty acid synthetase); *echA7* (Rv0971c) probable enoyl-CoA hydratase; (enoyl hydrase) (unsaturated acyl-CoA hydratase) (crotonase); *cdh* (Rv2289) probable CDP-diacylglycerol pyrophosphatase (CDP-diacylglycerol diphosphatase) (CDP-diacylglycerol phosphatidylhydrolase). The gene *papA3* (Rv1182) (encodes a polyketide synthase associated protein) has been reported to be involved in lipid metabolism, glycolipid assembly and possibly implicated in pathogenesis [27] was also expressed in *Mtb*:Δ*dosS* infected samples (Fig 3) and intraphagosomal *in-vitro* [23], TBDB database, http://www.tbdb.org). Although, we could not detect any lipid metabolism genes in *Mtb*:Δ*dosT* dataset, it has been suggested that host lipids are important sources of carbon and cholesterol required for ATP production in hostile environment [28, 29]. These observations support our previous findings [11] when numerous cholesterol clefts were present in mouse lungs infected with *Mtb* and mutant groups.

### PE/PPE family

The PE and PPE genes are unique to mycobacteria and are widely speculated to play a key role in tuberculosis pathogenesis [30, 31]. We examined these genes in all datasets. In WT *Mtb*, PPE26 (Rv1789) was induced to 1.8 fold, which plays an important role in protective immunity [32] while p27 (Rv2108, 1.8 fold induction) is in sync with Th1 response against *Mtb* infection [33]. Similarly PE15 (Rv1386, 1.9 fold induction) is involved in host-pathogen interactions that modulate innate immunity and mediate *Mtb* survival in macrophages [34]. In *Mtb*:Δ*dosR*, following genes were up regulated; Rv3812, 1.72 fold; Rv0915c, 1.75 fold; Rv1807, 1.85 fold; Rv1788, 1.94 fold; Rv3345c, 4.4 fold. It has been reported that antigen MBT41 encoded by Rv3812 induces Th1 immune response in C57BL/6 mice infected with *Mtb* [35] and Rv0915c encodes a protective antigen possibly involved in the early control of infection (‘Tuberculist’).

In *Mtb*:Δ*dosS*, we also detected PE/ PPE family genes. For example, Rv3873 (1.9 fold induction) plays an immunomodulatory role in regulating the pathophysiology of mycobacteria [34] and Rv3021 (induced to 2.0 fold) also expresses under hypoxia [36] and reaeration [37]. In *Mtb*:Δ*dosT*, Rv1790, a member of PE/PPE family of which function is unknown was upregulated.

### Regulatory proteins

We next examined the expression levels of two-component regulatory system genes implicated in bacterial virulence *in-vivo*. In *Mtb*, *senX3*-*regX3* two-component system is involved in the virulence [38, 39]. Amongst members of this regulon, transcriptional regulator genes Rv2488c, Rv3060c and Rv1267c were expressed in the lungs of mouse infected with wild type *Mtb* only. Rv3060c encodes a fatty acid metabolism regulator (FadR) probably known to regulate the isocitrate lyase (ICL), which may enhance the bacterial survival and persistence *in-vivo* [40, 41]. On the other hand Rv1267c (encodes EmbR) is involved in regulation of biosynthesis of the mycobacterial cell wall (Tuberculist). We also noted the expression of *mce3R* (Rv1963c, 1.8 fold induction), which plays a key a role in the adaptation and survival of *Mtb in-vivo* [42]. In *Mtb*:Δ*dosR*, several regulatory protein family genes, for example, Rv1129c (1.8 fold induction) required for intracellular growth in macrophages [43], Rv1151c (2.5 fold induction) required for intracellular cAMP signaling pathway [44] were detected. The cAMP signaling plays a role in the interaction of mycobacteria with macrophages during infection [45]. Similarly, Rv0890c induced to 2.3 fold is an Lrp/AsnC (leucine-responsive regulatory protein/asparagine synthase C) family transcriptional factor probably required for survival during persistence [46] and Rv1103c (1.6 fold induction) is involved in pathogenesis [47]. In *Mtb*:Δ*dosS* dataset, Rv2779c, an Lrp/AsnC transcriptional regulator known to be involved in various metabolic processes including starvation [48], Rv0328 (1.6 fold induction) is also reported to be expressed during growth in NRP state [24] and thioridazine [49]. In this part of analysis *pknJ* (Rv2088, 1.5 fold induction) encoding a transmembrane serine/threonine-protein kinase also expresses during hypoxia [36, 37], re-aeration [37] and intraphagosomal environment [23]. In particular, many of the genes required for bacterial persistence e.g. Rv2919c and Rv0744c [7], Rv2989 [50], Rv0405 [51], Rv3281 [52] etc., down-regulated in *Mtb*:Δ*dosS* dataset may indicate growth restriction in mouse lungs (Fig 2). These results also support our previous findings that *Mtb*:Δ*dosS* is attenuated in C3HeB/FeJ mice [11]. In *Mtb*:Δ*dosT* dataset, Rv0465c was induced whose expression is high following the nutritional stress [53] and Rv0386 encoding adenyl cyclase induced to 2.2 fold, is reported to be involved in virulence and may facilitate long-term intracellular survival of mycobacteria [54].

### Virulence, detoxification, adaptation

Various genes involved in virulence, detoxification and adaptation were examined next. In *Mtb*, *mce4A* (Rv3499c) required for persistent tubercular infection [55] was expressed to higher levels (1.8 fold induction). *mce4A* is a member of *kstR* (Rv3574) regulon and is involved in lipid catabolism [56]. Another gene *mce1A* (Rv0169) involved in host cell invasion by *Mtb* and survival in human macrophages [55] was expressed in *Mtb* dataset (1.65 fold induction). Similarly, induction of Rv1996 (1.8 fold), a member of DosR regulon may indicate the persistent infection [13, 57]. In *Mtb*:Δ*dosR* following genes; Rv3358c, Rv2757c, Rv2596 and Rv1103c which have not been studied in details but belonging to virulence, detoxification and adaptation functional category were upregulated. *In Mtb*:Δ*dosS*, vapC36 (Rv1982c) upregulated to 2.9 fold also reported to be induced under stress conditions such as diamide [58] and hypoxia [36]; vapB33 (Rv1241, 1.9 fold induction) also expresses under hypoxia [36] and inside macrophages [23]; vapb17 (Rv2526, 1.8 fold induction) also expresses under hypoxia [36], reaeration [37] and upon exposure to 0.05% SDS [59]; *ephG* (Rv2740, 1.6 fold induction), involved in detoxification following oxidative damage to lipids, is also shown to be expressed during non replicative persistence (bacteriostatic) [24]. In *Mtb*:Δ*dosT*, induction of Rv2190c (1.7 fold) indicates involvement in pathogenicity as it is required for full virulence of *Mtb* in mice [60] and Rv3417c, a chaperon associated with nucleoid [61], may play a role in DNA supercoiling, macromolecular crowding etc. required during hostile environment. *proV* (Rv3758c, 2.0 fold induction) mRNA levels were increased in *Mtb*:Δ*dosT* infected mouse lungs. ‘*proV’* is involved in osmoregulation as bacteria in the phagosome begin to grow and has been shown to increase during post-phagocytosis in cultured human macrophages [62] and in lungs of mice infected with *Mtb* [63].

### Pathways analyses in-vivo

To understand the functional relevance of mycobacterial genes expressed during infection, we used IntPath database [50] for pathway enrichment analysis and the enriched (over-represented) functional categories that are closely related to both pathogen growth and infection were compared. We were able to identify various pathways representing one or more functional category such as cell wall and cell processes, information pathways, intermediary metabolism, lipid metabolism, PE/PPE family, regulatory proteins and ‘virulence, detoxification, adaptation’ in all datasets (S2 Table). Most of these pathways were differentially expressed in *Mtb* vs. Dos mutants during growth in mouse lungs. For example, information pathways e.g. “DNA mismatch repair”, “RNA polymerase” (S1 Fig) and pathways belonging to lipid metabolism e.g. “Lipopolysaccharide biosynthesis”, "2-Oxobutanoate Degradation I", Carbon fixation pathways in prokaryotes, (S2 Fig) and intermediary metabolism pathways e.g. Antigen biosynthesis, sugar metabolism (glycolysis, gluconeogenesis, pyruvate phosphate pathways etc.), superpathways of chorismate, amino acid biosynthesis (S3 Fig) were significantly changed in mice lungs infected with *Mtb*. In *Mtb*:Δ*dosR*, pathways such as “Guanosine nucleotides de novo pathway of methionine biosynthesis, superpathway of amino acids biosynthesis, nucleotide biosynthesis, TCA cycle, NAD phosphorylation, etc. were significantly changed (S1–S3 Figs). On the other hand, *Mtb*:Δ*dosS* exhibited significantly different pathways changed in mouse lungs. Specifically serine-isocitrate lyase pathway, tryptophan degradation VII (via indole-3-pyruvate) tyrosine biosynthesis I, Inositol phosphate metabolism, glutamate metabolism, Taurine and hypotaurine metabolism, TCA cycle, nitrogen metabolism in addition to pathways related to metabolism and respiration etc. (S1–S3 Figs). The mutant *Mtb*:Δ*dosT* exhibited following pathways; arginine biosynthetic pathways, citrulline metabolism, enterobactin biosynthesis, urea cycle in addition to metabolic pathways, nicotinamide metabolism, (S1 and S3 Figs). The sulfur relay system pathway involved in cellular functions such as cell proliferation, apoptosis and DNA repair [64] was changed in both *Mtb* and *Mtb*:Δ*dosT*.

*Mtb* is able to grow on variety of carbon sources, but uses fatty acids as the major sources of carbon and energy essential for its growth during infection [65]. In summary, these pathways are closely related to TCA cycle (S1–S3 Figs), which is essential for the growth of *Mtb* growth and metabolism [65].

### Mycobacterial gene expression in *Mtb* and Dos mutants in mouse lungs

**Fig 4:**
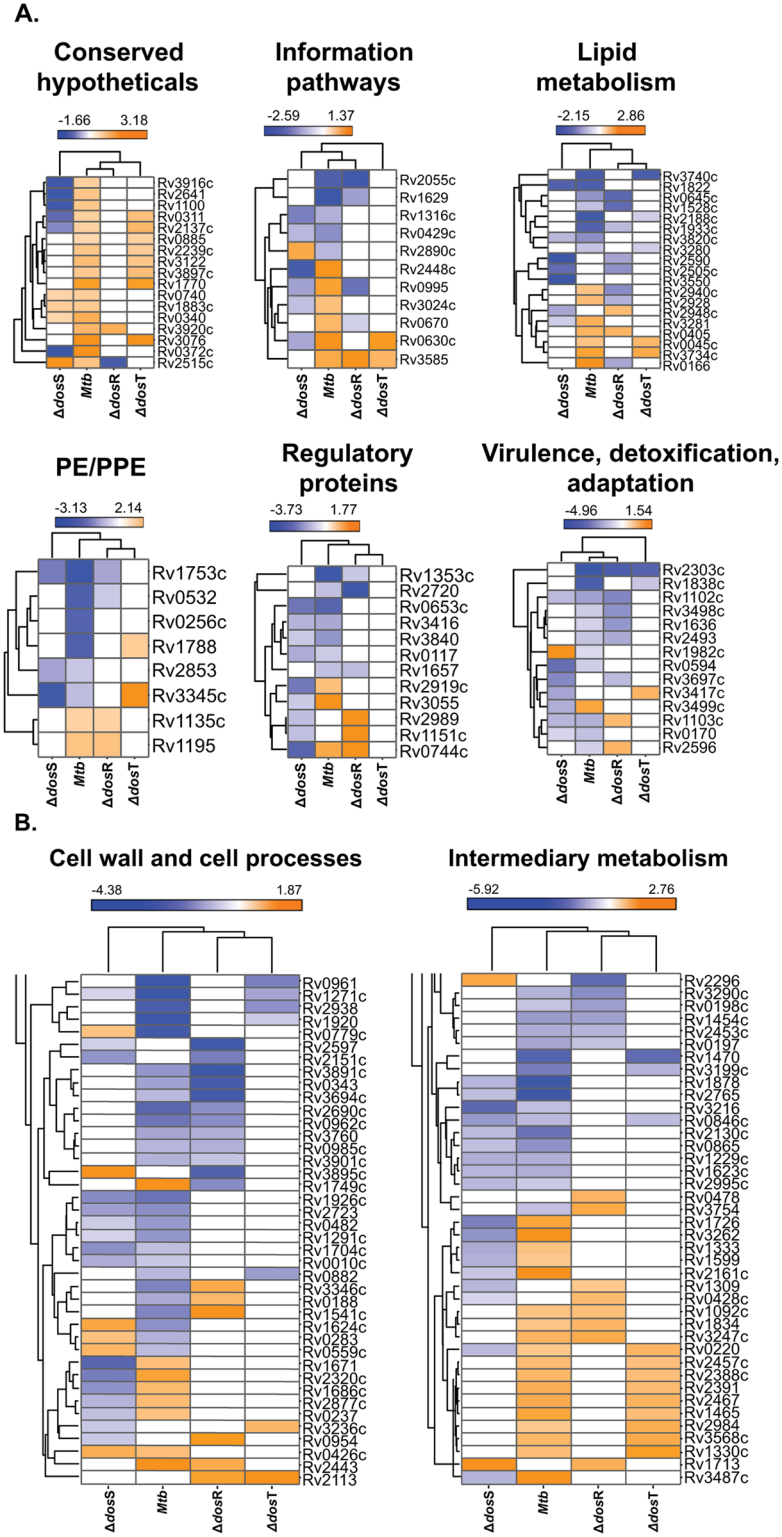
Hierarchical clustering of *Mtb* genes expressed in C3HeB/FeJ mouse lungs. Hierarchical clustering demonstrates the expression of common genes (low, blue to high, orange) in two or more datasets in C3HeB/FeJ mice. The data was compared to functional categories of *Mtb* genes described in the ‘Tuberculist’ database.

We also applied a hierarchical clustering algorithm [11, 66] to group the genes by expression patterns (down-, up-regulated or no-change in gene expression) that may reflect similar function once the bacilli establish the infection and persist in mouse lungs. Hierarchical clustering supported the gene classes belong to functional categories derived from ‘Tuberculist’ (Fig 4). We grouped eight such clusters from all four groups and these represent the genes highly expressed in more than one dataset, confirming their requirement in C3HeB/FeJ mouse lungs (this study) and BALB/c mice [7]. These clusters included the genes belonging to functional categories such as information pathways, lipid metabolism, immunomodulation, virulence, etc. required for survival or persistence. Following to gene expression pattern, the reduction in bacillary load of *Mtb*:Δ*dosS* in C3HeB/FeJ mouse lungs (Fig 2), ref. [11]) thus be explained and probably indicates the role of following genes in survival of bacilli; for example, the transcriptional regulator *glnB* (Rv2919c) required for survival of bacilli in mice [7] and macrophages [67] was up-regulated in *Mtb* but down-regulated in *Mtb*:Δ*dosS* infected samples indicate its importance in survival (Fig 2). Another gene, Rv2989 required for survival in macrophages [50] and during hypoxia [36] was down-regulated in *Mtb*:Δ*dosS* infected samples only (Fig 4). Similarly, Rv0744c which upregulates during hypoxia [36] and in mice [7] was downregulated in *Mtb*:Δ*dosS* samples (Fig 4), again indicates its role in *Mtb* survival. Similarly Rv0405 required for bacterial resistance in mice [51] and Rv3281 for growth and pathogenesis [52], were down-regulated in *Mtb*:Δ*dosS* infected samples. However, Rv0045c, a serine hydrolase enzyme possibly required for transition between dormant and active *Mtb* infection [26] and Rv0166 necessary for the persistence in murine model [68] were not altered in gene expression during *Mtb*:Δ*dosS* growth in mice (Fig 4).

The expression of Rv3585 gene encoding the DNA repair protein ‘RadA’ (Tuberculist) indicates that recombination of genes may occur during mycobacterial growth at these time points. We did not detect the ‘*radA’* levels in *Mtb*:Δ*dosS* dataset. DNA microarray analysis also lead us to the identification of variety of other genes that code for the proteins like putative transporters e.g. Rv0283, Rv2320c, Rv1686c etc. and membrane protein Rv1671, Rv0954, Rv0426c etc. whose expression were either upregulated or did not change during course of infection. The sigma factor *sigL* dependent transcription of Rv2877c that is believed to be important in *Mtb* pathogenesis [69] was also noted in *Mtb* WT dataset.

The genes belong to succinate metabolism are important for adaptation of *Mtb* to hypoxia [70]. For example DctA, a C4-dicarboxylate-transport transmembrane protein important for translocation of TCA cycle intermediates e.g. Succinate, fumarate or malate to *Mtb* were either upregulated (*Mtb* and *Mtb*:Δ*dosR*) or their expression levels were unchanged during course of infection in all dataset. Rv2443 levels have been shown to be upregulated during hypoxia *in-vitro* [24] and in mice [7].

At the same time different sets of genes whose function is conserved were expressed in all datasets e.g. members of the toxin-antitoxin system involved in virulence, detoxification and adaptation (vapB32/Rv1113, mce3R/Rv1963c, Rv0959, Rv3189 in *Mtb* WT); Rv3181c, vapB46/Rv3385c, Rv3749c, vapC21/Rv2757c, vapC40/Rv2596, mazE3/Rv1103c in *Mtb*:Δ*dosR*; vapC36/Rv1982c, vapC36/Rv1982c, vapB33/Rv1241, vapB17/Rv2526 in *Mtb*:Δ*dosS*; Rv0060, vapC13/Rv1838c in *Mtb*:Δ*dosT* (S1 Table). Similarly, genes changed in hypoxia *in-vitro* experiments [13, 24] were also detected in mouse lungs datasets (S3 Table). The hypoxia responsive genes may contribute to establishment of persistent infection during host environment. The upregulation of *dosR* regulon genes e.g. Rv0569, Rv1996, Rv0571c, Rv2004 in mouse lungs (S3 Table) indicate that bug experiences the stress such as hypoxia [13, 24] in hostile environment.

### Insights gained from comparison to previous genome-wide expression studies

**Fig 5:**
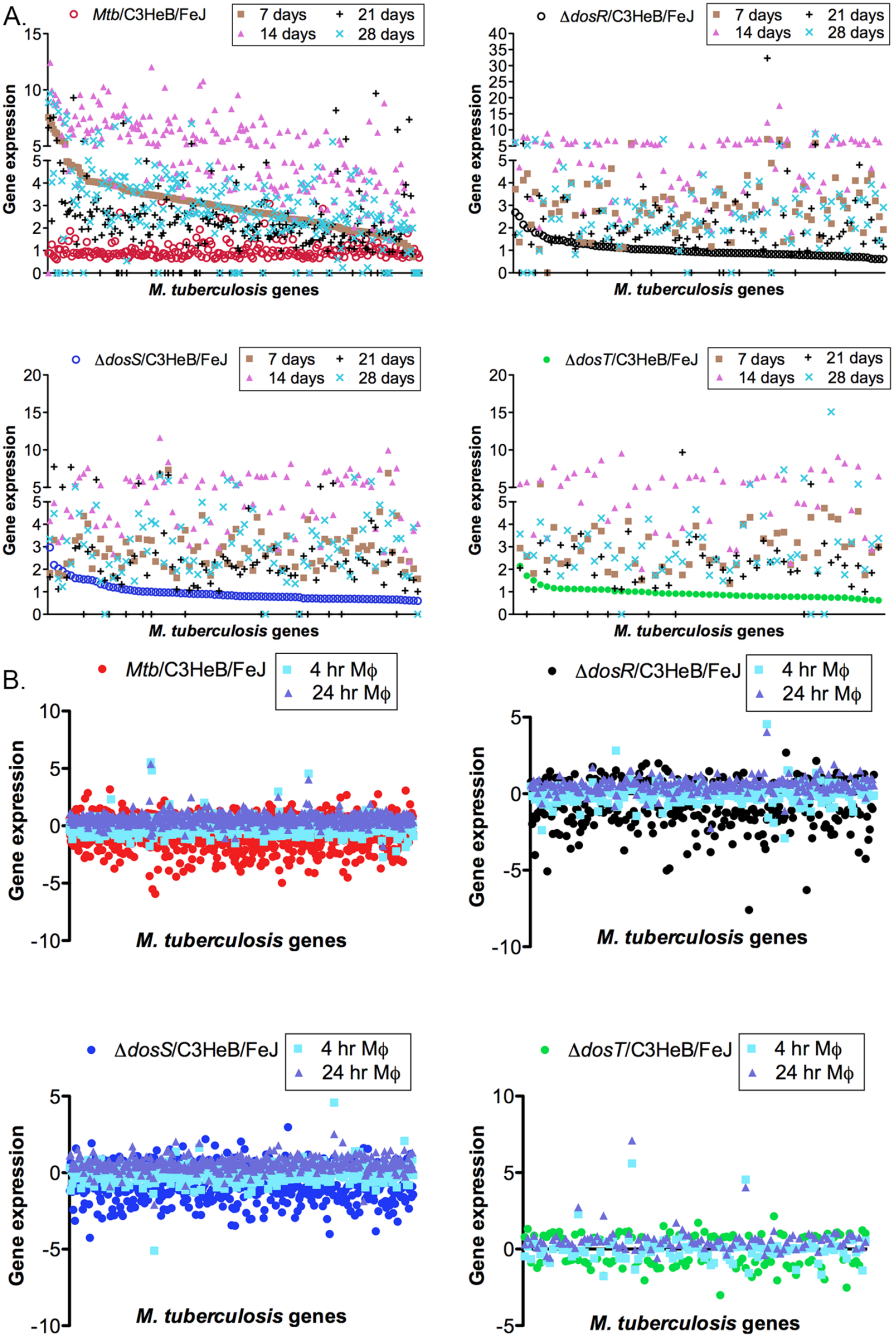
Scatter plot diagram showing similarity and dissimilarity in gene expression from various datasets. **A)**. Comparison of gene expression in C3HeB/FeJ mouse lungs infected with *Mtb* strains (red- *Mtb*; black- *Mtb*:Δ*dosR*; blue- *Mtb*:Δ*dosS*; green- *Mtb*:Δ*dosT*) versus gene expression profile in BALB/c mice [7] **B)**. Graph shows the bacterial genes and their expression levels in C3HeB/FeJ mouse lungs (this study) compared to infected macrophages at 4- and 24-hr post infection [23].

**Fig 6:**
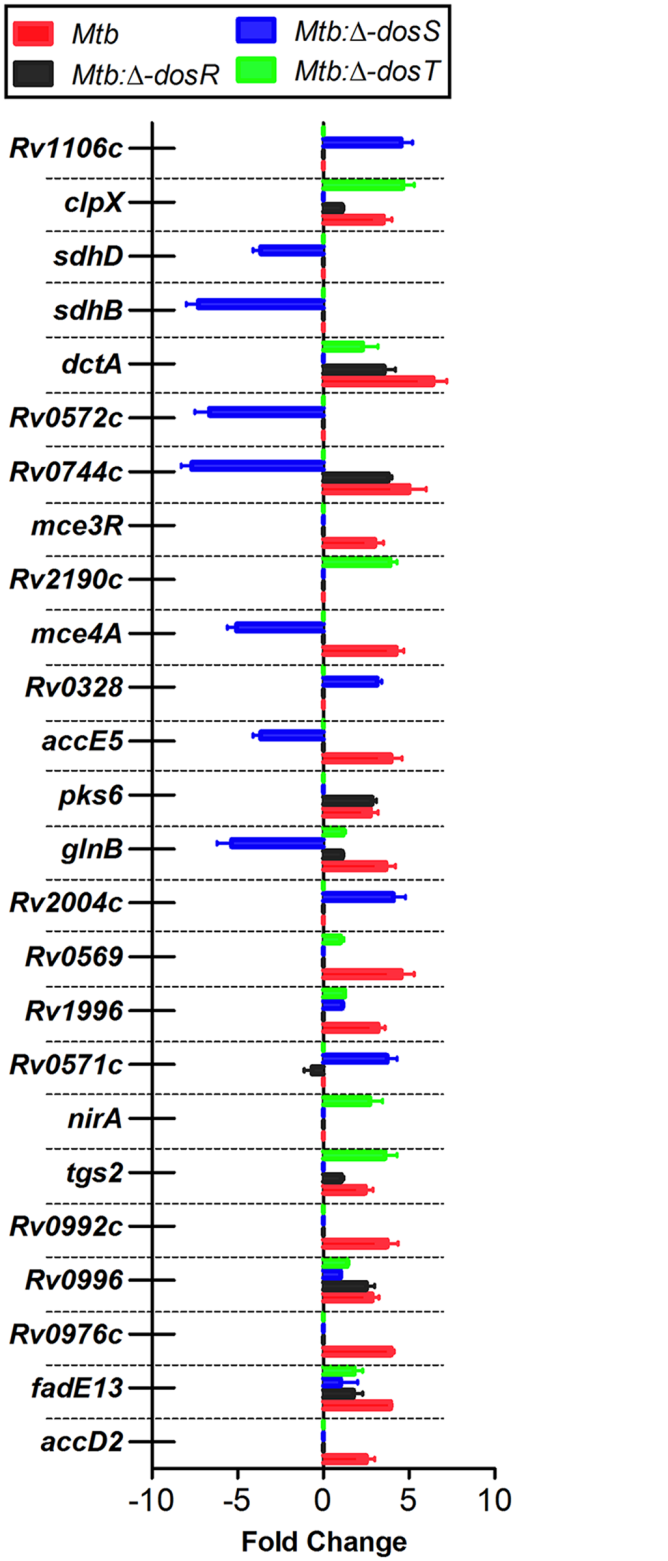
Validation of *Mtb* gene expression in mouse lungs by quantitative RT-PCR. The expression of indicated genes in intracellular bacteria was compared to that of bacteria growing exponentially in 7H9 broth by RT-PCR. The expression of each gene was normalized to *sigA* and fold change were calculated from three biological replicates.

**Fig 7:**
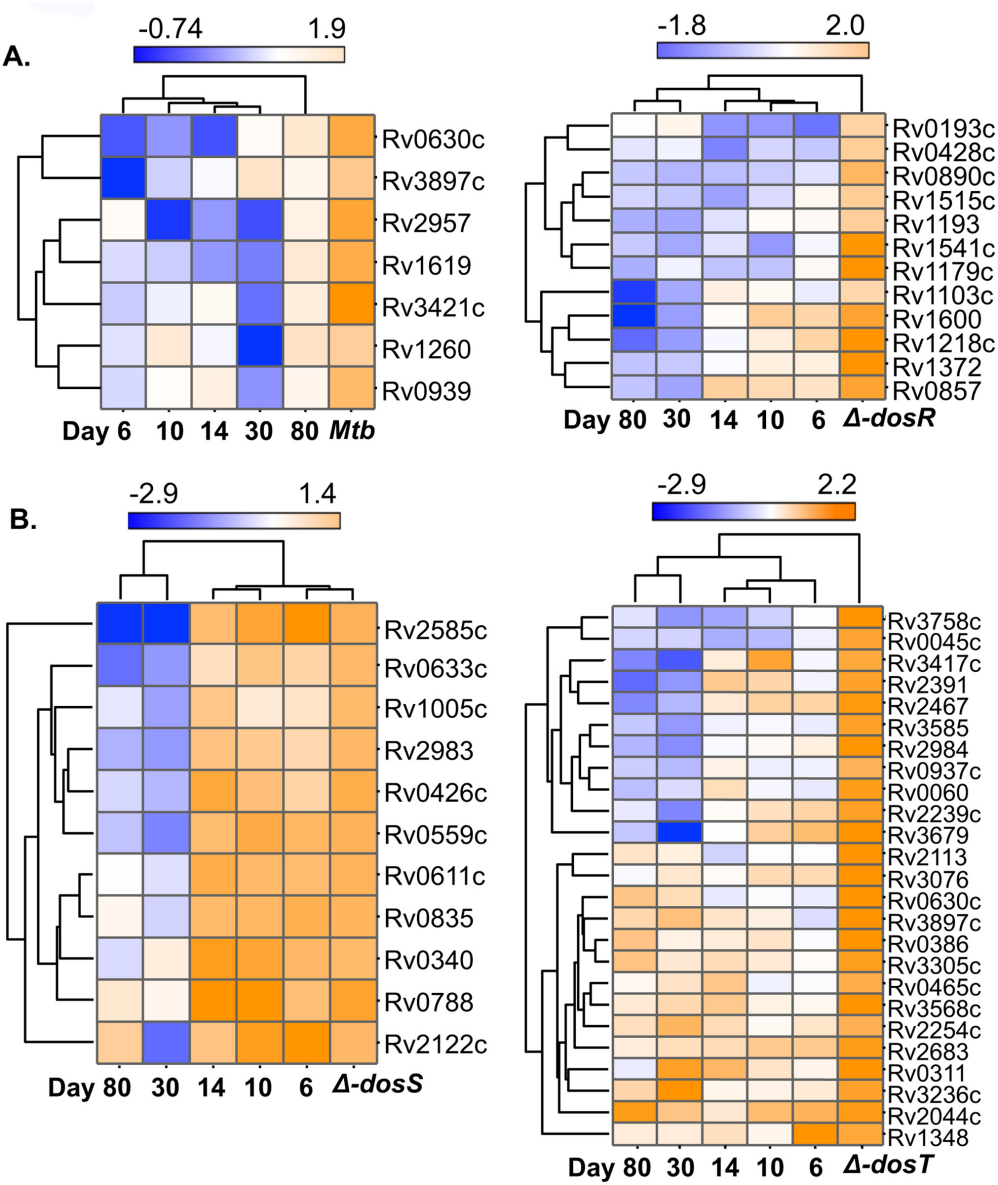
Hierarchical clustering of bacterial genes expressed in C3HeB/FeJ mouse lungs. A snapshot of few bacterial genes induced in C3HeB/FeJ mouse lungs upon infection with *Mtb* or *Mtb*:Δ*dosR* or *Mtb*:Δ*dosS* or *Mtb*:Δ*dosT* and their comparison with genes expressed during NRP [24] is shown. A gradual decrease or increase in color intensity indicates low (blue) or high (orange) expression. For example, a gradual increase in gene expression over 80 days of hypoxia indicates their requirement during both hypoxia *in-vitro* and chronic phase of infection in C3HeB/FeJ mouse lungs.

We performed a comparative transcriptomics analysis of our datasets (genes detected in chronic phase of infection) with data obtained from BALB/c mice (early time point) (Fig 5A), ref. [7], macrophages (Fig 5B), ref. [23] and *in-vitro* (NRP) conditions (Figs 6 and 7), ref. [24]. Functional grouping of genes based on gene expression profile demonstrated similarity or dissimilarity among various datasets (Figs 5 and 6 and S1, S3, S4, S5 Tables). Of note Rv0961, Rv0971c, Rv0966c, Rv0963c, Rv0974c, Rv0975c, Rv0976c and Rv0996 of iVEGI (*in-vivo* expressed genomic island) signature in BALB/c mice [7], were also detected in our datasets (Fig 5A and 6 and S4 Table).

Similarly, an overlap in genes C3HeB/FeJ mouse lungs (this study) vs. macrophages (Fig 5B and S5 Table), ref [23] indicates their requirement not only in macrophages *ex-vivo* but also *in- vivo*. For example, *clpX* gene required for stress tolerance [71] and bacterial growth [72] was upregulated in our datasets (S5 Table). An comparison with ‘NRP’ dataset [24] also showed an overlap of array of genes e.g. Rv2122c, Rv0045c, Rv0630c etc. (S3 Table). Rv2122c encodes a phosphoribosyl-ATP pyrophosphohydrolase, required for the growth of *Mtb* and is a member of *ideR* (iron-dependent regulator), probably involved in virulence [73]. The transition between dormant and active *Mtb* infection requires reorganization of lipid metabolism and activation of a battery of serine hydrolase enzymes such as Rv0045c [74]. We found upregulation of Rv0045c in our datasets that might indicate transition in state of bacilli within host. The perturbation of Rv0630c indicates modification of *Mtb* genes in hostile environment [54]. Similarly, upregulation of *radA* (Rv3585, encodes a DNA repair protein) and Rv3417c expressions indicate DNA modifications during *in-vivo* growth (Tuberculist) and interactions with Toll like Receptors respectively [75] (S3 Table). Hierarchical clustering demonstrated an overlap in genes expressed on C3HeB/FeJ mouse lungs (this study) vs. *in-vitro* (NRP) conditions (Fig 7). We grouped the time points NRP day-6, -10, -14, 30, and -80. We considered the time point NRP day 80 since this represents long-term hypoxia. A gradual increase in gene expression over 80 days of hypoxia indicates their requirement during both hypoxia *in-vitro* and chronic phase of infection in C3HeB/FeJ mouse lungs. In *Mtb*:Δ*dosR*, the upregulation of Rv0890c, Rv048c, Rv1515c, Rv1218c etc. in C3HeB/FeJ mouse lungs and gradual decrease in expression of these genes over 80 days of hypoxia indicates their requirement in chronic phase of infection only. Similarly in *Mtb*:Δ*dosS*, a gradual decrease in expression of Rv2585c, Rv0633c, Rv1005c, Rv2122c etc. over 80 days of long-term hypoxia indicate their requirement in chronic phase of infection only. In *Mtb*:Δ*dosT* both, upregulated (Rv3236c, Rv0311, Rv0465c, Rv2044c etc.) and down-regulated (Rv0045c, Rv3758c, Rv0937c, Rv0060 etc.) genes over 80 days of hypoxia indicate differential gene expression compared to C3HeB/FeJ mouse.

The comparative analysis reveals an overlap in many of the genes and their expression levels between C3HeB/FeJ and BALB/c mice, which clearly indicates that these genes are required during both early [7]) and chronic phase (this study) of infection. Similarly, an overlap in gene expression between C3HeB/FeJ and macrophage or NRP conditions indicated their requirement both during *in-vivo* and *in-vitro*.

## Discussion

The outcome of the host-pathogen interactions is in large part shaped by selective gene expression during infection [76]. Thus, bacterial gene expression during course of infection has the potential to provide specific and key knowledge about the physiology og the pathogen within its intra-granulomatous niche. It is conceivable that this information will generate a list of *in-vivo* druggable targets of chemotherapy, which may be otherwise ignored.

Thus, here, we describe the gene expression profile of *Mtb* strains viz. *Mtb*, *Mtb*:Δ*dosR*, *Mtb*:Δ*dosS* and, *Mtb*:Δ*dosT* in the human-like lung lesions of C3HeB/FeJ mice. In particular, *Mtb*:Δ*dosS* is primarily focused. The data presented here provide important new information about the adaptation of this pathogen inside the host with co-expression of similar (Fig 4) or unique (Fig 3) genes being detected among these groups. Thus, for example, the co-expression of following genes viz. Rv2488c, Rv3060c, Rv1129c, Rv0890c, Rv0328, Rv2088, Rv0465c in the functional category ‘response regulator’ highlights their importance in regulating gene expression during *in-vivo* growth. Specifically Rv0465c encodes a transcription factor designated RamB [77], which is a key regulator of isocitrate lyase and glyoxylate shunt, a metabolic pathway critical for *Mtb* persistence [78]. The expression of RamB appears to be regulated by the SigE/SigB regulatory axis [77], which is itself regulated by SigH [22, 58, 79], PknB [80], ClgR [81, 82] and other regulatory loops, many of which are known to be induced during macrophage infection as well as *in-vivo* [23, 83]. The SigH/SigE/SigB/ClgR regulatory circuit is critical for the pathogen to face the host oxidative burst and required for initial infectivity in primate lungs [84]. It appears that this network is responsible for evasion of antibacterial responses leading to the prolonged survival of *Mtb* [85].

Similarly, Rv2088 encodes for a protein kinase PknJ, which is also induced in the lungs of guinea pigs infected with *Mtb* [86]. Among the various targets that PknJ is experimentally known to phosphorylate and activate [87], EmbR is a transcriptional factor required for the expression of the embCAB operon, that encodes the critical cell wall arabinosyl transferases [88]. This pathway is important for both the acquisition of resistance to ethambutol (a frontline antimycobacterial drug) and the cell wall Lipoarabinomannan/Lipomannan ratio (that plays a key role in immune-evasion). Another known PknJ target is the methyl transferase MmaA4/Hma, which is involved in mycolic acid biosynthesis.

The expression of the transcription factor Rv0328 also appears to be governed by the SigH/SigE/SigB axis [23, 49] indicating a role in both immune evasion and pathogenesis. Rv1129c encodes a transcription factor that is essential for the induction of the propionyl-CoA assimilating methyl citrate cycle enzymes [43], which are required for both intra-phagosomal survival of *Mtb* as well as for survival on cholesterol-containing media (which is a key carbon source for *Mtb* during intra-phagosomal persistence in lungs [89], as well as for the catabolism of cholesterol [43]. Rv1129c is also known to be dependent upon SigE for its expression [77]. Rv2488c encodes a transcription factor that is also known to be induced in guinea pig lungs [86], and is predicted to be in the SenX3/RegX3 network [38], critical for defense against damage to DNA, which is experienced by *Mtb* during oxidative stress *in-vivo* [90, 91]. Thus, transcriptomics analysis of *Mtb* derived from human-like caseous lungs lesions in C3HeB/FeJ mice at chronic stage paints a picture where the pathogen experiences diverse stress conditions including but not limited to oxidative stress, hypoxia, adaptation to less-preferred carbon and nitrogen sources aka cholesterol, and damage to cell-surface, DNA and lipids. Hence, gene-expression modules controlled by these regulators represent important *in-vivo* targets.

Lipid metabolism plays a key role in the *Mtb* pathogenesis during which *bacilli* use fatty acids as a sole carbon source for the survival *in-vivo* [43]. In addition, cell wall lipids play variety of roles in physiology and pathogenesis during infection [92]. Many of the genes involved in lipid metabolism, critical in the cell membrane biosynthesis, sugar metabolism, bacterial resistance within host, survival, immunomodulation and pathogenesis could be detected in all datasets. This strongly suggests the requirement of modified lipid metabolism in-vivo, as has been postulated and studied by others [26, 93, 94]. Recently we have reported that *Mtb*:Δ*dosS* is attenuated in Kramnik mice [11] we, therefore, predicted that the attenuation of *Mtb*:Δ*dosS* mutant may results from lack of expression of bacterial genes required for survival and persistence during infection. Following genes required for survival in mice and macrophages were downregulated in *Mtb*:Δ*dosS* dataset; *glnB* (Rv2919c) [7, 67], Rv2989 [50] Rv0744c [7, 51] Rv2989 [50], Rv0405 (38) and Rv3281 [52].

Several other genes highly expressed *in-vivo*, including genes that were shown to be involved in cell wall biosynthesis (e.g. Rv1350, Rv3840c, Rv3895c etc.), transcriptional regulation (e.g. Rv2488c, Rv2799c, Rv0329 etc.) may contribute to the establishment of the infection inside the host. Moreover, we detected differentially expressed genes in all datasets (96–98% genes were unique). A set of 650 genes in *Mtb*; 255 genes in *Mtb*:Δ*dosR*, 406 genes in *Mtb*:Δ*dosS*, 114 genes in *Mtb*:Δ*dosT* (Fig 1) were expressed in C3HeB/FeJ mice with at least 36 and not more than 53 genes were common in all datasets. The pathways significantly changed in *Mtb*, *Mtb*:Δ*dosR*, *Mtb*:Δ*dosS* or *Mtb*:Δ*dosT* during growth in C3HeB/FeJ mice lungs were information-, intermediary-, and lipid metabolism-pathways (S1–S3 Figs). Genes such as *fdxC* (Rv1177) induces at low pH, DNA damage stress [95], and during growth in macrophages [96]. The list of the genes in our datasets (S4 and S5 Tables) and those induced in macrophages [23] and BALB/c mice [7] suggests that the host immune response after infection is characterized by macrophage activation.

A change in the growth-dependent genes and their expression levels e.g. those belonging to information pathways and intermediary metabolism (different genes but from the same functional category among strains, Fig 3) was observed. An analysis of the transcriptional response of *Mtb* genes in Kramnik mice observed in the present study suggests that protective functions are conserved which could facilitate the adaptation of *Mtb* in hostile environment. In summary, transcriptomics analysis of *Mtb* and Dos mutants also indicates both the macrophage-like and multiple stress environments that may influence the adaptation and affect the persistence of bacilli intracellularly.

## Conclusions

As part of this study we present our analysis of i) *Mtb* gene expression at the chronic stage of infection in the C3HeB/FeJ mouse model and ii) present comparisons with the various mutants in the hypoxia-sensing regulon controlled by the DosR transcription factor. The expression of iVEGI genes [7] in our datasets indicates their requirement not only during onset (and the early stages) of *Mtb* infection but also their significant contribution during the chronic stages. In addition, our analysis identified both core gene sets and core categories which were present in all datasets, as well as specific genes which correlate with the relative attenuation of the *Mtb*:Δ*dosS* mutant in this mouse model. Several of these genes are important for lipid metabolism as well as for survival in the wake of diverse host-generated stress conditions such as hypoxia, oxidative stress, DNA damage, lack of availability of preferred carbon sources etc. These findings have the potential to allow us to better understand the dynamics of bacilli in C3HeB/FeJ mice that mimic the pathology of human lung granuloma and may provide the information for possible drug and/ or vaccine targets. Further, more in-depth studies are required to better understand as to how these gene signatures correspond to bacterial virulence or control of infection.

## Ethics Statement

All the animal samples used as part of this study were generated in a previous study, which was entirely approved, by the Tulane National Primate Research Centre (TNPRC) Institutional Animal Care and Use Committee (IACUC) as a protocol, which was submitted by the Principal Investigator.

## Supporting Information

S1 Fig Integration of functional category ‘information pathways’ and its group percentage. The results obtained are based on an overlap between the total numbers of genes changed in each of the biological replicate of mice lung samples to the genes in a functional category assigned in Tuberculist. These numbers were then used to calculate group percentage for functional category ‘information pathway’, changed in *Mtb* or Dos mutants in mouse lung using IntPath [50].
(TIF)

S2 Fig Integration of functional category lipid metabolism and its group percentage. The results summarize the group percentage for functional category ‘lipid metabolism pathways’ based on an overlap between the total numbers of genes changed in each of the biological replicate of mice lung samples to the genes in the functional category ‘lipid metabolism’ assigned in Tuberculist.
(TIF)

S3 Fig Integration of functional category intermediary metabolism and its group percentage. The group percentage was calculated based on an overlap between the total numbers of genes changed in each of the biological replicate of mice lung samples to the genes in functional category ‘intermediary metabolism’ assigned in Tuberculist.
(TIF)

S1 Table Functional categories and their genes changed in *Mtb-* or Dos mutants-infected mouse lung samples. The Table summarizes genes identified in various functional categories based on the information available in the ‘Tuberculist’ database for *Mtb* H37Rv genome.
(XLS)

S2 Table Enriched biological pathways changed in mouse lungs. Various pathways representing one or more functional category as per Tuberculist and IntPath [50] database are shown.
(XLS)

S3 Table List of genes associated with bacterial persistence. The Table summarizes hypoxia responsive genes changed in C3HeB/FeJ mouse lungs (this study) versus *in-vitro* conditions [24].
(XLS)

S4 Table Bacterial gene expression in C3HeB/FeJ mouse lungs. Comparison of genes and their expression in C3HeB/FeJ mice lungs infected with *Mtb* strains (*Mtb*, *Mtb*:Δ*dosR*, *Mtb*:Δ*dosS*, *Mtb*:Δ*dosT*) versus genes expressed in *Mtb* infected BALB/c mice lungs [7].
(XLS)

S5 Table Biological pathways changed in *Mtb* or Dos mutants infected mouse lungs. *The Table summarizes enriched pathways significantly changed (P<0.05) in mouse lungs infected with *Mtb* or *Mtb*:Δ*dosR* or *Mtb*:Δ*dosS* or *Mtb*:Δ*dosT* relative to *in-vitro* grown cultures. The ‘p-value’ for a pathway is based on IntPath database that uses hyper-geometric test to find most significant pathways in an input gene list to the number of genes assigned for a functional category in the genome [50].
(XLS)
