# Supplementary figures and images for: In-Vivo Gene Signatures of Mycobacterium tuberculosis in C3HeB/FeJ Mice
Source: PLoS One. 2015 Aug 13;10(8):e0135208. doi: 10.1371/journal.pone.0135208 (PMC4535907; doi:10.1371/journal.pone.0135208)

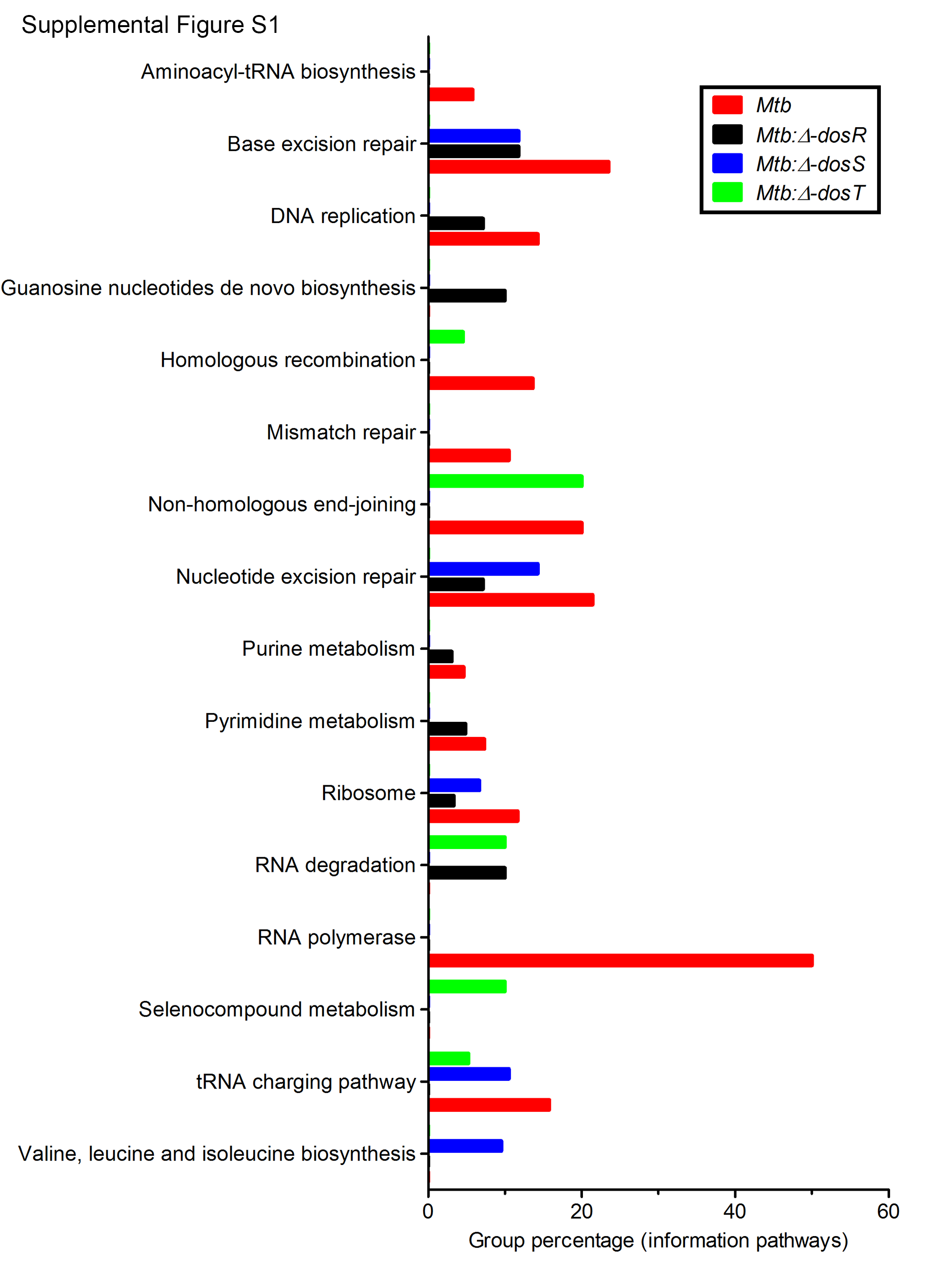

Supplement: S1 Fig — The results obtained are based on an overlap between the total numbers of genes changed in each of the biological replicate of mice lung samples to the genes in a functional category assigned in Tuberculist. These numbers were then used to calculate group percentage for functional category ‘information pathway’, changed in Mtb or Dos mutants in mouse lung using IntPath [50]. (TIF) [file pone.0135208.s001.tif]

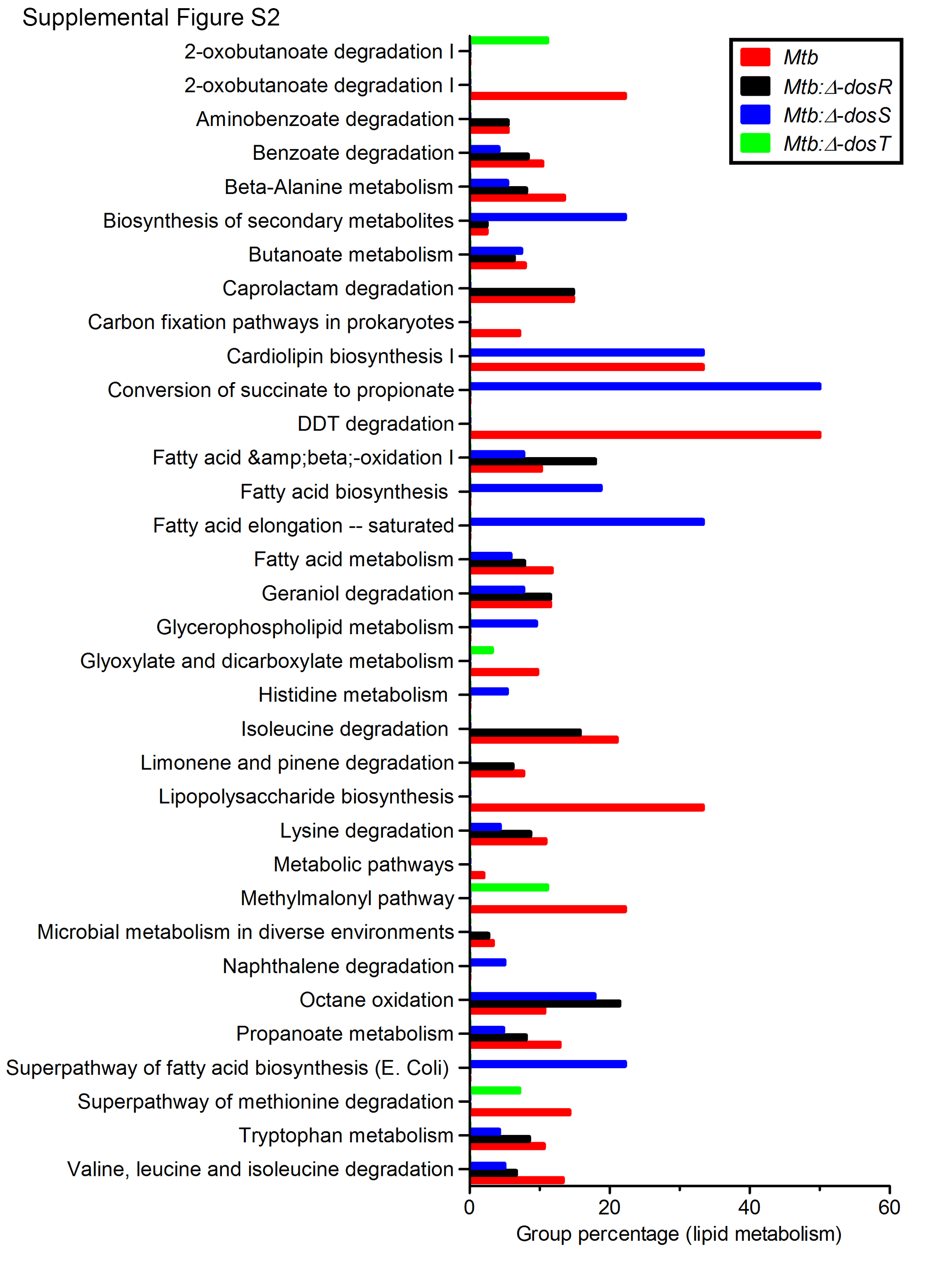

Supplement: S2 Fig — The results summarize the group percentage for functional category ‘lipid metabolism pathways’ based on an overlap between the total numbers of genes changed in each of the biological replicate of mice lung samples to the genes in the functional category ‘lipid metabolism’ assigned in Tuberculist. (TIF) [file pone.0135208.s002.tif]

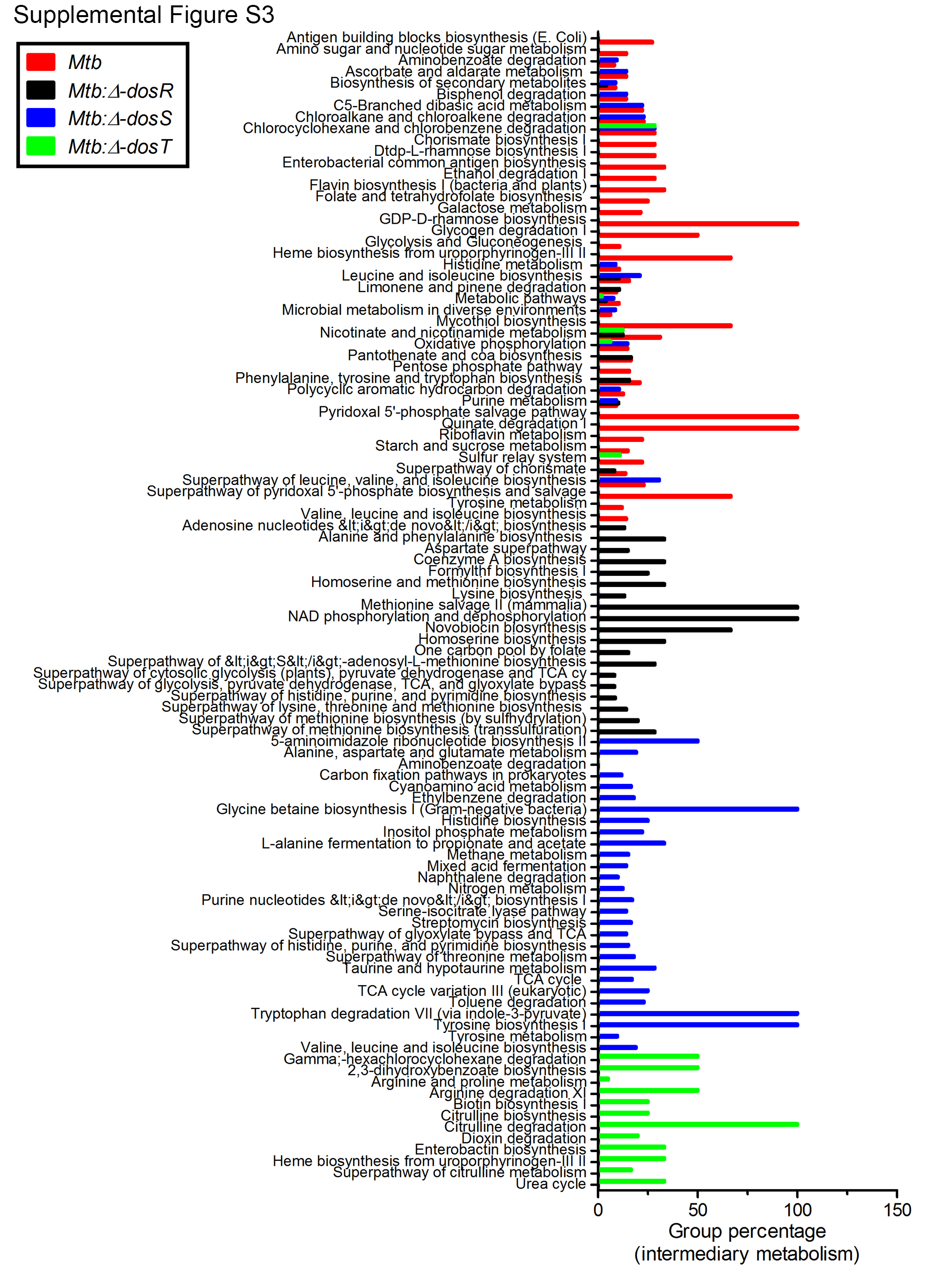

Supplement: S3 Fig — The group percentage was calculated based on an overlap between the total numbers of genes changed in each of the biological replicate of mice lung samples to the genes in functional category ‘intermediary metabolism’ assigned in Tuberculist. (TIF) [file pone.0135208.s003.tif]
